# Knowledge, attitude and practices related to visceral leishmaniasis among residents in Addis Zemen town, South Gondar, Northwest Ethiopia

**DOI:** 10.1186/1471-2458-13-382

**Published:** 2013-04-24

**Authors:** Agersew Alemu, Abebe Alemu, Nuraini Esmael, Yared Dessie, Kedir Hamdu, Biniam Mathewos, Wubet Birhan

**Affiliations:** 1 School of Biomedical and Laboratory Sciences, College of Medicine and Health Sciences, University of Gondar, Gondar, Ethiopia

**Keywords:** KAP, Visceral Leishmaniasis, Addis Zemen

## Abstract

**Background:**

Visceral leishmaniasis (VL), commonly known as kala-azar is a systemic disease caused by parasitic protozoan species of genus *Leishmania* and transmitted by species of *Phlebotomus* (sand flies). It is a poverty-related disease and associated with malnutrition, displacement, poor housing, weakness of the immune system and lack of resources. For the success of prevention and control programs of any disease, the most important prerequisite is community participation. Therefore, this study was aimed to assess the knowledge, attitude and practice of residents towards VL in Addis Zemen town, south Gondar, Northwest Ethiopia.

**Methods:**

Community based cross-sectional study was conducted among residents in Addis Zemen town from February to March 2012. A total of 346 households were selected by using simple random sampling techniques from three kebeles in the town. Data was collected using structured Questionnaire. For knowledge, attitude and practice variables each right response was given a score of 1 while a wrong or unsure response was scored 0. Data were double entered and analyzed using SPSS-15 statistical software. The frequency distribution of both dependent and independent variables were worked out.

**Results:**

From a total of 346 study participants (136 males and 210 females), 87.6% heard of the disease kala-azar. From participants who heard about kala-azar 93.5% males and 86.7% females had awareness about the disease. The majority (95.7%) of participants had favourable attitude towards the treatment of kala-azar whereas 14.8% didn’t use anything to prevent it. More than half of the respondents (68.6%) did practice proper methods for the prevention and control of kala-azar in the study area.

**Conclusion:**

In general our findings showed that the residents had good awareness and favourable attitude about the disease, but their overall practice about prevention and control of the disease was low. Therefore, our investigation call for continued and strengthened behavioral change communication and social mobilization related activities.

## Background

Visceral leishmaniasis, commonly known as kala-azar, is a systemic disease caused by parasitic protozoan species of genus *Leishmania*. It is a chronic, systemic disease characterized by fever, hepato splenomegaly, lymphadenopathy, pancytopenia, weight loss, weakness and, if untreated, death [1]. The ethological agents belong to the *Leishmania donovani* complex, *L.d donovani, L.d infantum* and *L.d arachibaldi* in the Old world and *L.d chagasi* in the New world. The Old world species are transmitted by species of the genus *Phlebotomus* (sandflies). Human, wild animals and domestic dogs are known to act as reservoir hosts, the parasite enters macrophages, where it multiplies and establishes the infection [2].

Currently, leishmaniasis occurs in four continents and is considered to be endemic in 88 countries, 72 of which are developing countries [3]. Nineteen percent of all visceral leishmaniasis cases occur in Bangladesh, Brazil, India, Nepal and Sudan [3-5]. In the horn of Africa, VL has focal distribution in two distinct ecologic settings: 1) the semi-arid regions in the North where phlebotomies oriental’s breeds in cracks in black cotton clay soil; and 2) the savannah and forest areas in the South where the vectors *P. martini* and *P. celiae* are found in association with macro terms termite in mounds [6,7].

In Ethiopia, long recognized VL endemic foci are situated in Metema and Humera along the border with Sudan in the Northwest, reflecting the first ecologic pattern, and in regions of Lake Abaya, Omo River and the Aba Roba plains in the South, following the second pattern, with an estimated country-wide incidence of 2,000 cases per year [8,9]. The Libo Kemkem district, where the first VL outbreak occurred, is in the Northwest of Ethiopia. This district has never been endemic to VL until the recent epidemic of April 2005. The outbreak claimed a large number of lives before its cause was identified. The outbreak started at least as early as 2004, but was initially misdiagnosed as malaria [10,11].

Visceral leishmaniasis is often found in areas that are remote, with absent or undeveloped health facilities, where tools for screening and identification of patients are inadequate, and above all with no or few trained man power. Due to lack of updated information, even the most critical cases remain untreated or unreported, and can represent a reservoir of infection (mainly in areas where transmission is from man to man) for family members and neighbors [12].

For the success of prevention and control programs of any disease, the most important prerequisite is community participation. Cooperation of the affected population is essential in the implementation and use of program activities. Program implementers need to understand the disease-related knowledge, attitude, and practices of the community, because these are the important determinants of community participation. Despite increased prevalence of VL in different parts of Ethiopia due to different reasons like HIV coinfection, antimonials drug efficacy and others [13-17], there is no study conducted regarding KAP of community towards VL in Ethiopia and few studies have been conducted in other parts of the world [18-24]. Therefore, this study was aimed at assessing the knowledge, attitude and practice of residents towards VL in Addis Zemen, a town located in the endemic area of Libo Kemkem district, Northwest Ethiopia.

## Methods

### Study area and design

A community based cross-sectional study was conducted from February to March 2012 among settlers in Addis Zemen town, south Gondar, northwest Ethiopia. Addis Zemen is the capital of Libo Kemkem wereda (district), in the Amhara Region of northwestern Ethiopia (average altitude 2,000 m above sea level)*.* The district consists of 32 kebeles with an estimated population of 19, 878 (from Addis Zemen city administration) in 2004. Addis Zemen is located between Bahir Dar and Gondar on the major road connecting Addis Ababa to the Red Sea, crossing known foci of intense VL transmission in Metema. The district has one health center and most VL patients treated at this health center lived in the Libo Kemkem and Fogera districts.

### Sample size and sampling techniques

The sample size was calculated using the WHO recommended statistical formula for health studies, n = Z2 P (1-P)/d2 where, n = number of study subjects (households) enrolled in the study, Z = test statistic which allows to calculate our result with 95% confidence (1.96), d = the level of precision (5%) and P = proportion (prevalence) to be used on estimates which was expressed in decimal (so increase our sample size we used maximum p = 0.5). Since the total households in the town was less than 10,000 we used the following correction formula (nf = ni/(1 + ni/N)) where N = total households of the town (3533). For each of the 3 kebeles, the households number was selected by using probability proportion (ni = NiXn/N), where ni = total number of study subjects in each kebele, Ni = total number of households in each kebele, n = total number of study subjects obtained and N = total number of households in Addis Zemen town. Therefore a total of 346 households (HH) (from kebele 0l, 115 HH, from kebele 02, 89 HH and from kebele 03, 142 HH) were included in our study.

Households were selected from each kebele by using simple random sampling. The list of households for each kebele, knowing that the list did not contain any hidden order was obtained from the Kebele leaders and was used as a sampling frame. Simple random sampling method was employed to select households from each kebele from household registry using a table of random numbers. Household heads of each randomly selected household who lived for at least six months in the town were included and when the selected household was inconvenient the households before or after the indicated one was sampled for replacement.

### Data collection

The study instrument was an interviewing questionnaire which was comprised of four parts. Part A related to study subjects sociodemographic background, Part B on knowledge regarding VL, Part C on attitude scale towards VL, and Part D on practice related to VL prevention. The knowledge, attitude and practice questionnaire was modified from the instrument used by other countries for knowledge, attitude & practice studies on VL. Knowledge was assessed using a 8-item questionnaire which includes knowledge on VL, attitude was assessed using a 6-item questionnaire and the questions on practice had 5 items related to prevent sand fly bite. The English questionnaire was translated into simple Amharic (local language) and back translated into English. Pre- test of questionnaire was done on 5% and the result was used to improve the phrasing of questions in the questionnaire. Questionnaire validation tests showed that the Alpha Cronbach was 0.86 for knowledge, 0.72 for attitude and 0.74 for practice.

### Scoring

For knowledge, each correct response was given a score of 1, while a wrong or unsure response was scored 0. Total knowledge scores can range between 0–8. Knowledge scores from 0 to 4 were considered as poor knowledge, while knowledge scores more than 4 were considered as good knowledge regarding VL. Attitude towards VL was assessed using a 6-item questionnaire: attitude scores between 0 to 3 was considered as negative, whereas scores from 3 to 6 were considered as positive. Practice was assessed using a 5-item questionnaire, and a report of more than 2 was considered as good practice for VL control.

### Data management and analysis

During data collection process, the data were checked for completeness and all incomplete or misfiled questions were sent back for correction. Data were double entered and analyzed using SPSS-15 statistical software (SPSS Inc. Chicago, 2007). Descriptive statistics were used to give a clear picture of background variables like age, sex and other variables in well structured questionnaire. The frequency distribution of both dependent and independent variables were worked out.

### Ethical consideration

This community-based study was carried out after ethical clearance obtained from the University of Gondar College of Medicine and Health Science, School of biomedical and laboratory sciences. After discussing the purpose and method of the study, written permission was sought from concerned government officials before data collection. Prior to interview, the questioners were asked for their willingness to participate in the study.

## Results

### Socio-demographic characteristics of study subjects

A total of 346 individuals were involved in this study, 136 (39.3%) of the respondents were males and 210 (60.7%) were females. The mean age of participants was 35 year. More than half of the participants (188, 54.3%) were married, 109 (31.5%) were government employees and orthodox Christianity was the dominant religion 282 (81.5%) in the area. Regarding their level of education 118 (34.1%) participants were unable to read and write.

The majority of participants 335 (96.8%), were Amhara by ethnicity. Regarding the year of residency, the majority 312 (90.2%) lived in the area since more than 3 years (Table 1).

**Table 1:** Socio demographic characteristics of study participants in Addis Zemen town, South Gondar, Northwest Ethiopia, 2012

**Variables**		**Frequency**	**Percentage (%)**
Age	18-25	44	12.7
	26-32	95	27.5
	33-42	98	28.3
	> 42	109	31.5
Sex	Female	210	60.7
	Male	136	39.3
Marital status	Single	74	21.4
	Married	188	54.3
	Widowed	34	9.8
	Divorced	50	14.5
Occupation	Farmer	27	7.8
	Government employee	109	31.5
	Student	33	9.5
	House wife	77	22.3
	Others	37	10.7
Religion	Orthodox	282	81.5
	Muslim	57	16.5
	Protestant	6	1.7
	Others	1	0.3
Educational status	Unable to read and write	118	34.1
	Only read and write	38	11.0
	Elementary	43	12.4
	Secondary school	42	12.1
	12 and above	105	30.3
Ethnicity	Amhara	335	96.8
	Tigre	5	1.4
	Others	6	1.7
Year of Residency	< 1 years	8	2.3
	1-2 years	26	7.5
	> 3 years	312	90.2

### Knowledge on VL among study subjects

Among the total participants, 303 (87.6%) had heard of the disease, 182 (60.1%) knew that the disease is infectious, and 128 (68.1%) responded that sand fly bite is the main way of transmission. The majority of the respondents (293, 96.7%) knew that if the disease is left untreated the outcome will be death. More than half (188, 62%) knew more than one sign and symptom of the disease, and 57 (18.8%) said that abdominal swelling was the only sign and symptom of the disease. From the 123 male participants who heard about kala-azar, 115 (93.5%) were knowledgeable; on the other hand, from the 180 female participants who heard about kala-azar, 156 (86.7%) were knowledgeable. Generally, according to scoring results, 271 (89.4%) participants were knowledgeable (Table 2).

**Table 2:** Knowledge on VL among study participants in Addis Zemen town, South Gondar, Northwest Ethiopia, 2012

**Variables**			**Frequency**	**Percentage (%)**
Heard about kalazar n = 346	Yes		303	87.6
	No		43	12.4
Infectiousness of the Diseases n = 303	Yes		182	60.1
	No		50	16.5
	I don’t know		71	23.4
Mode of transmission n = 182	Malaria mosquito		1	.5
	Worms		4	2.1
	sleeping with infected person		18	9.6
	Sand fly		128	68.1
	I don’t know		27	14.4
	Others		10	5.3
Sign and symptoms n = 303	Fever		3	1.0
	Fatigue		1	0.33
	Abdominal swelling		57	18.8
	I don’t know		53	17.5
	Others		1	0.33
	More than one answer		188	62
Preventability of disease n = 303	Yes		246	81.2
	No		16	5.3
	Don’t know		41	13.5
Out come if left untreated n = 303	Death		293	96.7
	Self cure		2	0.7
	Don’t know		8	2.6
Knowledge on VL (overall)	Knowledgeable	Male	115	42.4
		female	156	57.6
	Total		271	**89.4**
	Not Knowledgeable	Male	8	25
		Female	24	75
	Total		32	**10.6**

### Attitude towards VL among participants in Addis Zemen town

From the total of 303 respondents who heard about the disease, the majority (290, 95.7%) have positive attitude towards the treatment of kala-azar. Regarding their treatment preference the majority (286, 94.4%) preferred to get treatment at health facilities. Two hundred sixty two (86.4%) respondents said that a complete cure from the disease is possible. More than 75% of the respondents (239, 78.9%) were of the opinion that controlling kala-azar by community participation is possible. More than half of the respondents (161, 53.1%) believed that kala-azar is a health problem in Addis Zemen town and the surrounding kebeles. Overall, 264 (87.1%) of respondents have favorable attitude towards (Table 3).

**Table 3:** Attitude towards VL among study participants in Addis Zemen town, South Gondar, Northwest Ethiopia, 2012

**Variables**		**Frequency**	**Percentage (%)**
Treatability of the disease n = 303	Yes	290	95.7
	No	8	2.6
	I don’t know	5	1.7
health problem in the community n = 303	Yes	161	53.1
	No	132	43.6
	I don’t know	10	3.3
Community participation to prevent VL n = 303	Yes	239	78.9
	No	23	7.6
	I don’t know	41	13.5
Treatment preference n = 303	Traditional healer	6	2
	Health center	286	94.4
	Holy water	11	3.6
Complete cure of the disease n = 303	Yes	262	86.4
	No	22	7.3
	I don’t know	19	6.3
Patient Care n = 303	Cleanliness	26	8.6
	Bed net	17	5.6
	Isolation of patient	41	13.5
	Precaution in diet	5	1.6
	I don’t know	24	7.9
	More than one answer	190	62.7
Attitude (overall)	Positive attitude	264	87.1%
	Negative attitude	39	12.9%

### Practice of respondents towards VL prevention and control in Addis Zemen town

From the total 303 who heard about the disease, 25 (14.8%) didn’t use any method to prevent kala-azar. The majority (284, 93.7%) have at list one bed net. Regarding work time preference when the temperate is high, 42 (13.9%) preferred night time. More than half of respondents (208, 68.6%) were practiced properly for the prevention and control of leishmaniasis (Table 4).

**Table 4:** Practice of respondents towards VL prevention and control in Addis Zemen town, South Gondar, Northwest Ethiopia, 2012

**Variables**		**Frequency**	**Percentage (%)**
Prevention of sand fly n = 167	Bed net	33	19.8
	DDT	6	3.6
	Cleanliness	3	1.9
	Isolation of patients	1	0.6
	Not use any prevention methods	25	14.8
	I don’t know	20	11.8
	More than one answer	79	47.4
Use of bed net n = 303	Yes	284	93.7
	No	19	6.3
Sleeping outdoor n = 303	Yes	32	10.6
	No	271	89.4
Sleeping condition n = 32	Under tree shade with bed net	6	18.7
	Under tree shade without bed net	26	81.3
Work time Preference when temperature is high n = 303	Day time	151	49.8
	night time	42	13.9
	Both	110	36.3
Practice (overall)	Good practiced	208	68.6%
	Not Good practice	95	31.4%

## Discussion

Visceral leishmaniasis is known to prevail in undetermined magnitude in the various localities of Ethiopia. In the area where the epidemiology of VL has been soundly established, the disease is considered to be alarming, contributing to about a third of the crude mortality rate in the absence of provision for early diagnosis and treatment [25].

The result of our study showed that most of the respondents (87.4%) have heard about kala-azar and 89.4% of them were knowledgeable. This result is lower than that from a study conducted in East Africa (Kenya and Uganda) where 95% participants have heard of kala-azar [22]. The variability between studies might be due to a lack of community health education, community awareness, socioeconomic status of the different areas, and the fact that kala-azar is a recently established disease in the Libo Kemkem district.

The fact that kala-azar is an infectious disease and can be transmitted from one person to another person was known by 60.1% of the respondent, whereas 23.4% of the respondents didn’t know its infectiousness, and 86.4% knew that a complete cure of the disease is possible. Sixty eight percent of the participants said that the causative agent of the disease was transmitted through sand fly bite and 14.4% of the respondents didn’t know about the mode of transmission. This result is higher than that found in Sudan [21] where only 6% indicated that the disease is transmitted by sand fly bite. This might be due to the disease outbreak in 2005 which helped the community to get more information and educational status different between the two areas.

Seventeen percent of the respondents had no idea of the sign and symptoms of the disease. This is similar to a study conducted in rural areas of Bihar state India (16.1%) [19]. More than half of the respondents (62%) in our study knew at least more than one sign and symptoms of the disease. The majority of the participants (96.7%) knew that if the disease is left untreated the outcome will be death, and only 0.7% of the respondents said that the outcome will be self cure. People’s knowledge about the outcome of the disease is high; this might be due to an increased attention towards leishmaniasis in Addis Zemen health center after the outbreak in 2005 and/or role of health extensions in teaching the community currently.

More than three forth of the respondents (81.2%) said that preventability of the disease is possible, only 5.3% of the respondents said that the disease couldn’t be prevented and the rest (13.5%) didn’t know whether it could be prevented or not. People’s knowledge about the preventability of the disease is high. This might be due to the fact that as people knows about the preventability of malaria (the fact that both are vector-borne diseases), they would conclude that leishmaniasis can also be prevented.

When the overall attitude of the study subjects is taken into account, 87.1% had a favorable attitude towards transmission and prevention of VL. The majority of the respondents (95.7%) were aware that the disease can be treated, while only 2.6% believed that it can’t be treated at all. This result is higher than that of a study conducted in rural areas of Nepal, where 78.9% (Titaria) and 48.8% (Haraincha) were aware that the condition can be treated, while less than 2% believed that it cannot be treated at all [18]. This might be due to the outbreak in 2005 [26], the Addis Zemen health center and Medicines sans frontiers-Greece gives special attention to diagnosis and treatment of kala-azar, allowing people to know about the treatability of the disease, or can be due to difference in the settings (Addis Zemen is an urban area while the study in Nepal was conducted in rural areas) and in time between the two studies.

The majority (86.4%) of the respondents believed that a complete cure of the disease is possible, and only 7.3% believed that it can’t be cured completely. Therefore, people’s attitude about the complete cure of the disease is high. This might be due to different reasons like community awareness, and the people’s tradition to ask patients, which helps to know more about kala-azar. Approximately 80% of the respondents believed that kala-azar could be controlled through community participation, whereas 7.6% of the respondents didn’t believe. Only few of the respondents (3.6%) preferred to seek treatment from Holy Water, whereas 94.4% of the respondents preferree to seek for treatment from health facilities. This result is similar to that from a study conducted in a highly endemic rural area of India (95%) [20].

In the present study it was found that 68.6% of the respondents practiced well, while 31.4% of the respondents didn’t practice well for the prevention and control of the disease. For the prevention of sand fly bites, 19.8% of the respondents use only bed nets, 3.6% only DDT, while 14.8% of the respondents didn’t use any prevention methods against sand fly bites. A large majority (93.7%) of the respondents used bed nets. This result is higher than those from rural areas of Nepal; where 58% of villagers in Titaria and 36.8% in Haraincha used bed nets [18], and rural areas in Bihar state India (23.9%) [19]. This might be due to the fact that the government gives bed nets to people for the control and prevention of malaria in this area of Ethiopia, or to differences in time of investigation, in the socioeconomic status of the people, in people’s awareness and to the fact that Addis Zemen is an urban area.

Ten point six percent of the respondents slept outdoors in farms, and 18.7% of them used bed nets while sleeping outdoors. Thirteen point nine percent of the respondents used to work at night when the temperature is high, but approximately half of the respondents (49.8%) still preferred to work during the day even with high temperatures. This might be due to socioeconomic status of the population, low electrical light supply, and people’s tradition to work at day time.

Thus to avert the spreading of disease to areas that are non-endemic for kala-azar in Libo Kemkem district, the results of this study emphasize the need for increasing awareness activities through the involvement of health workers, and of the school in the community on a massive scale. Therefore, this is the first study in Ethiopia that used probability sampling techniques and provided baseline information for further studies. However, it should be noted that this study was not supported by qualitative methods.

## Conclusion

In general our findings showed that people are knowledgeable about the disease, but knowledge about transmission, sign and symptoms and the infectious origin of the disease was still not very high. Concerning disease control, the people’s attitude towards complete cure of the disease, treatability of the disease and control of the disease through community participation were favorable. Even though the people’s knowledge about the disease was good, their overall practice about prevention and control of the insect vector (sandflies) indicates that there is still a gap in implementation of their knowledge. Therefore, our investigation calls for continued and strengthened behavioral change communication and social mobilization related activities.

## Competing interests

The authors declare that they have no competing interests.

## Authors’ contributions

AA: conceived the study, undertook statistical analysis and drafted the manuscript. AA, KH*,* YD*,* NE, BM and WB: initiated the study undertook statistical analysis and has major contribution in drafting the manuscript. All authors contributed to the writing of the manuscript and approved the submitted version of the manuscript.

## Pre-publication history

The pre-publication history for this paper can be accessed here:

http://www.biomedcentral.com/1471-2458/13/382/prepub
